# Overcoming collinearity in path analysis of soybean [*Glycine max* (L.) Merr.] grain oil content

**DOI:** 10.1371/journal.pone.0233290

**Published:** 2020-05-22

**Authors:** Murilo Viotto Del Conte, Pedro Crescêncio Souza Carneiro, Marcos Deon Vilela de Resende, Felipe Lopes da Silva, Luiz Alexandre Peternelli

**Affiliations:** 1 Departamento de Fitotecnia, Universidade Federal de Viçosa, Viçosa, Minas Gerais, Brasil; 2 Departamento de Biologia, Universidade Federal de Viçosa, Viçosa, Minas Gerais, Brasil; 3 Empresa Brasileira de Pesquisa Agropecuária (EMBRAPA), Centro Nacional de Pesquisa de Florestas, Colombo, Paraná, Brasil; 4 Departamento de Estatística, Universidade Federal de Viçosa, Viçosa, Minas Gerais, Brasil; Federal University of Mato Grosso do Sul, BRAZIL

## Abstract

Path analysis allows understanding the direct and indirect effects among traits. Multicollinearity in correlation matrices may cause a bias in path analysis estimates. This study aimed to: a) understand the correlation among soybean traits and estimate their direct and indirect effects on gain oil content; b) verify the efficiency of ridge path analysis and trait culling to overcome colinearity. Three different matrices with different levels of collinearity were obtained by trait culling. Ridge path analysis was performed on matrices with strong collinearity; otherwise, a traditional path analysis was performed. The same analyses were run on a simulated dataset. Trait culling was applied to matrix R originating the matrices R_1_ and R_2_. Path analysis for matrices R_1_ and R_2_ presented a high determination coefficient (0.856 and 0.832, respectively) and low effect of the residual variable (0.379 and 0.410 respectively). Ridge path analysis presented low determination coefficient (0.657) and no direct effects greater than the effects of the residual variable (0.585). Trait culling was more effective to overcome collinearity. Mass of grains, number of nodes, and number of pods are promising for indirect selection for oil content.

## Introduction

Soybean has been very important to Brazilian agribusiness. The oleaginous crop comprises approximately 20% oil by weight [1] and this product has been used in several applications, especially as kitchen oil and biodiesel. The crop is an important source of oil and ranks first in oil production in the world scenario [2] and the global demand for soybean oil is arising [3].

Quantitative traits, such as soybean oil, protein content, and grain yield are quantitative traits [4, 5], and are therefore controlled by a large number of genes, undergo a strong environmental influence, and usually show low heritabilities. Thus, the indirect selection is recommended to achieve genetic gains [6]. The viability of indirect selection is commonly investigated by correlations studies [7]. However, [8] emphasize that correlations among traits are purely a measure of association and do not investigate the cause-and-effect relationships and according to [9] the association between two traits can be influenced by a third trait or a group of them. [10] proposed path coefficient analysis, which is a multiple regression expanded and allows unfolding the correlation coefficients of direct and indirect effects on a basic variable [11]. Path Analysis was first used in plants by [12] and it was later used in other crops.

The estimation of the path analysis coefficients can be adversely affected by the multicollinearity effects among traits. Multicollinearity occurs when the observation of the independent variables, or their combinations, are correlated [11, 14]. When two variables are highly correlated it becomes hard to individually estimate the relations of each explanatory variable no the main variable because they contribute colletively to explain linear relations [14]. [13] advise performing the multicollinearity diagnostic on the correlation matrix of explanatory variables, since in presence of multicollinearity the variances associated to the path coefficients estimators may become too large and leads to unreliable estimates [14, 15]. In presence of multicollinearity, [12] recommend using ridge regression, which can obtain the solution to the vector parameters, even with significant multicollinearity, by the sum of small constant values (k) in the diagonal of the correlation matrix, as proposed by [16, 17]; or by excluding variables highly associated as presented by [18].

Many studies regarding path analysis on soybean that aimed to study the effects of traits on grain yield are available in the literature. [15, 19–24]. These studies show that it is usual to find high correlations among traits and, consequently, multicollinearity. Thus, methods to overcome multicollinearity must be studied, since most of these papers were not concerned to compare different methods for overcome collinearity.

Furthermore, despite of the large number of scientific papers about path analysis in soybean, no papers concerned grain oil content is found. Thus, the current study aimed to: a) understand the correlation among soybean traits as well as estimate its direct and indirect effects on oil content; b) verify the efficiency of ridge path analysis and trait culling to overcome colinearity problems.

## Material and methods

### Experimental conduction

The experiment was installed in 2016, February 22, in the Soybean Genetic Breeding Program’s greenhouse of the Plant Science Department of the University Federal of Viçosa, Viçosa, Minas Gerais.

The experimental design was a randomized block with 5 replicates. Since the pots filled all tables of the greenhouse, we arranged the treatments in blocks in order to account possible environmental gradient. The treatments consisted of 34 F_1_ soybean populations originated from a partial diallel and 15 commercial presented in S1 Appendix according to National Cultivar Registration [25]. Those cultivars used as genitors comprised between 5.5 and 9.0 [26]. The data of commercial cultivars were the ones used for statistical analysis. The plots consisted of two pots containing two plants of the same treatment (genotype). Sowing was performed in a substrate composed of three parts soil, two parts sand, and one part of organic substrate. The greenhouse was adapted using lamps in order to adjust the photoperiod and provide an appropriate environmental since the photoperiod is critical as the flowering inductor in soybean [27].

The experiment was carried in accordance with the recommendations of [28]. Twelve vegetative, productive, and grain quality traits were evaluated in each plant as they reached the appropriate phenological stage, according to [29] as described in Table 1.

**Table 1:** Code, description and assessment methodology of traits evaluated in soybean plants.

CODE	DESCRIPTION	ASSESSMENT METHODOLOGY
DF	Days to Flowering	from emergency stage (VE stage) until beginning bloom (R_1_ stage)
HF	Height of plants on Flowering	from the base until the last apical meristem at R_1_ stage
DM	Days to Maturity	measured from the VE stage until full maturity (R_8_ stage)
HD	Hypocotyl Diameter	measured 2 centimeters below the cotyledon node at R_8_ stage
HM	Height of plants on Maturity	measured from the base to the last apical meristem at R_8_ stage
NP	Number of Pods per plant	number of pods per plant measured at R_8_ stage
NN	Number of Nodes per plant	number of nodes in the main stem per plant measured at R_8_ stage
NB	Number of Branches per plant	number of lateral branches per plant measured at R_8_ stage
NG	Number of Grains per plant	measured at R_8_ stage
MG	Mass of Grains per plant	corrected to 13% of humidity
OC	Oil content of grains	measured in percentage of soybean intact grains at R8 stage and corrected to 13% of humidity
PC	Portein content of grains	

### Genetic-statistical analysis

The genetic parameters were estimated and the genetic values predicted by analyzing only the genitors’ information. The statistical analyses were performed with the genetics and statistics software SELEGEN-REML/BLUP as described by [30, 31], using the following statistical model:

y=Xr+Zg+e,where: (1)

Where **y** is an (*gr*) x 1 vector of response variable, being *g* and *r* the number of genotypes and replicates, respectively; **r** is an (*r*) x 1 vector of unknown fixed effects fixed effects of replicates; **g** is an (*g*) x 1 vector of random effects of genetic values; and **e** is an (*gr*) x vector of random effects of error. **X** (*g x r)* and **Z** (*r x g)* are the incidence matrix.

The significance of the random effect was obtained by the likelihood ratio test by the analysis of deviance as recommended by 30. The analysis of deviance is derived from the difference between the deviance of the complete model and reduced model missing the effect which must be tested using chi-squared test with one degree of freedom [30, 32].

After obtaining the genetic values, we estimated the correlations between the traits. Then, a multicollinearity diagnosis in the original correlation matrix (matrix R) composed of 11 explanatory traits was performed. The multicollinearity diagnosis was performed considering the condition number as stated by, therefore, the matrices were classified as with weak, moderate, or strong collinearity for condition numbers lower than 100, between 100 and 1000, and greater than 1000, respectively [32].

**Fig 1:**
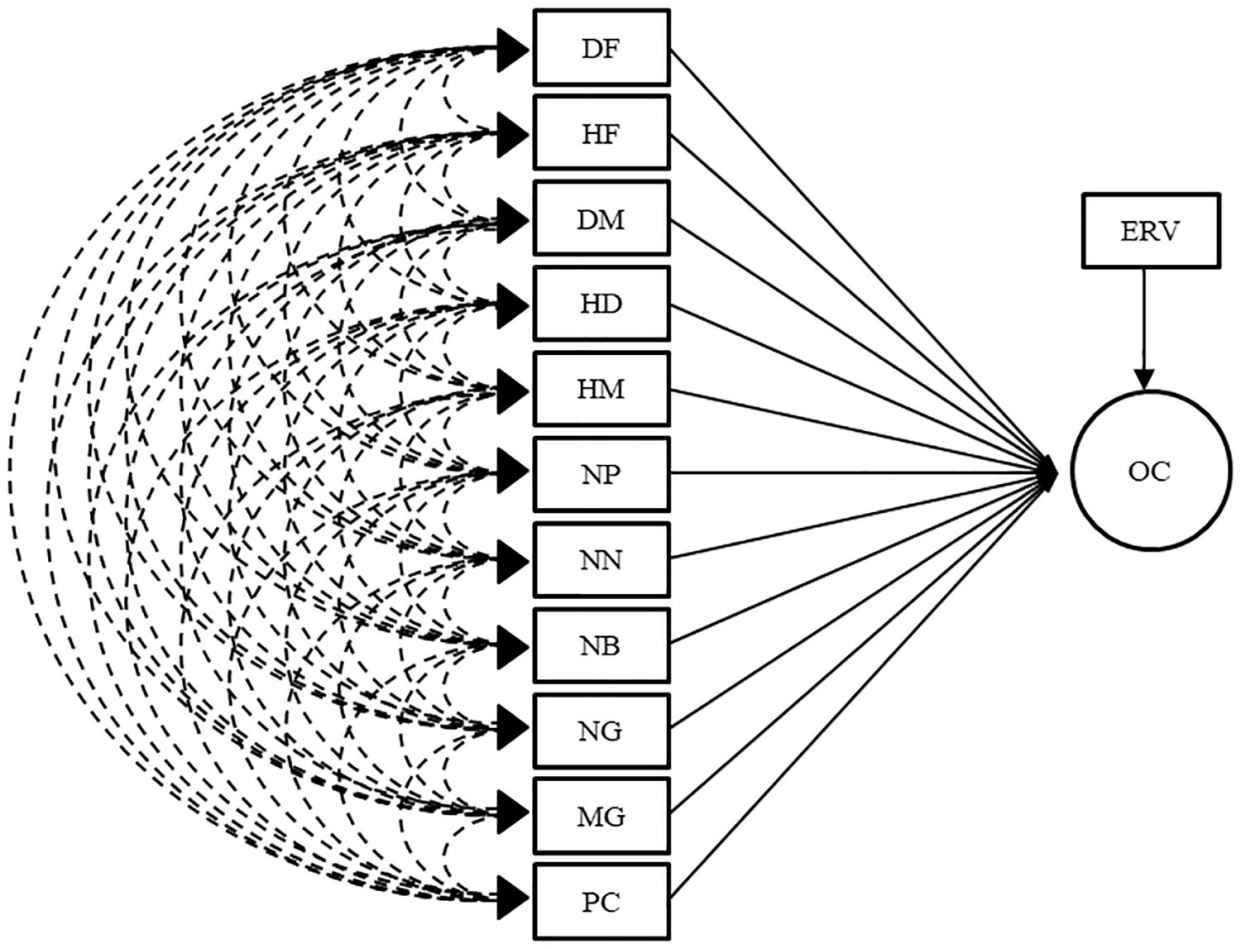
Path analysis diagram for matrices R and Rs’s. Oil content was considered the main trait and DF—days to flowering; HF—height of plants on flowering; DM—days to maturity; HD—hypocotyl diameter; HM—height of plants on maturity; NP—number of pods per plant; NN—number of nodes in the main stem per plant; NB—number of branches per plant; NG—number of grains per plant; MG—mass of grains per plant; PC—protein content in grains; OC—oil content in grains and ERV–effect of residual variable, the explanatory variables.

**Fig 2:**
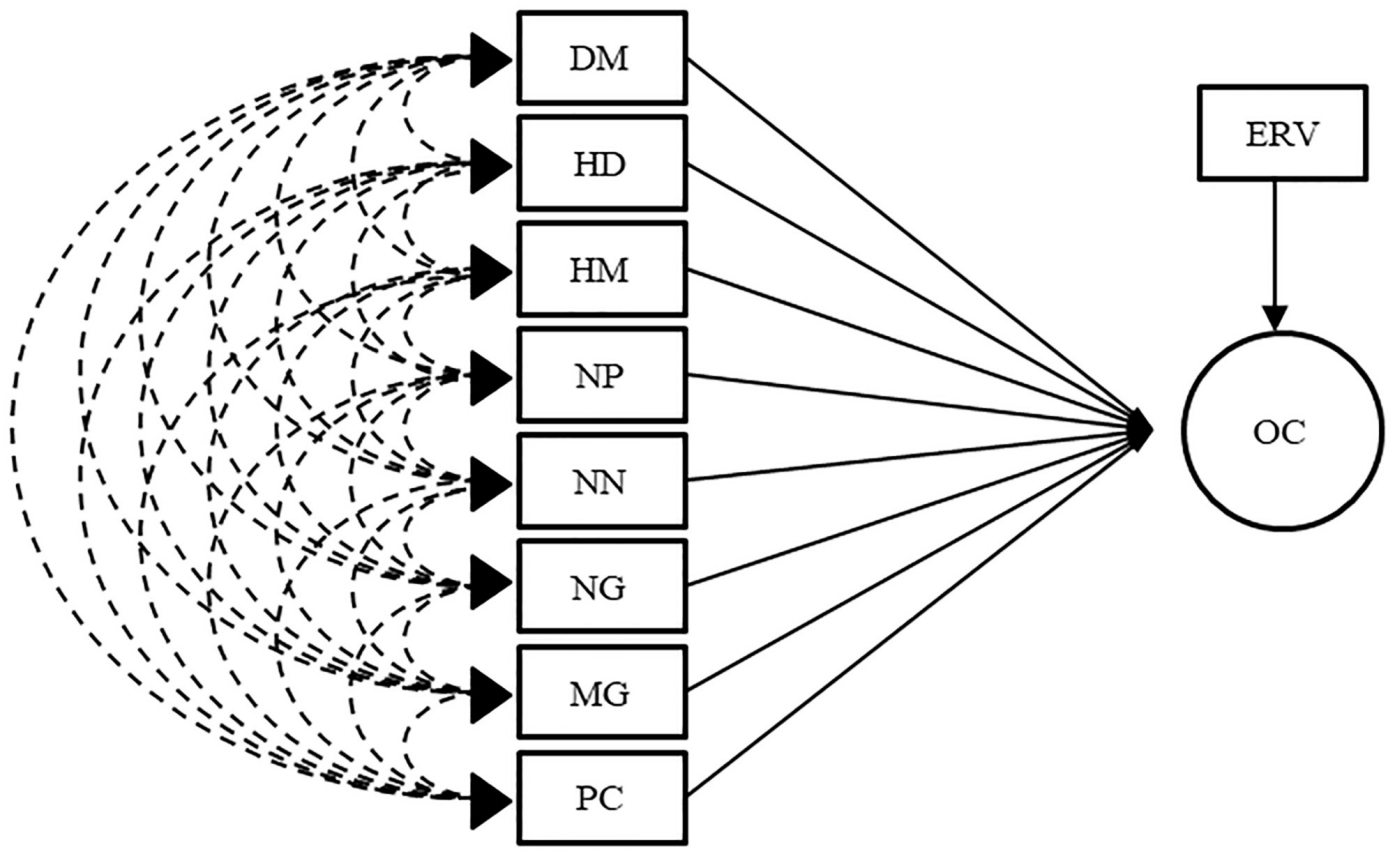
Path analysis diagram for matrices R_1_ and R_1_s’. Oil content was considered the main trait and DM—days to maturity; HD—hypocotyl diameter; HM—height of plants on maturity; NP—number of pods per plant; NN—number of nodes in the main stem per plant; NG—number of grains per plant; MG—mass of grains per plant; PC—protein content in grains; OC—oil content in grains and ERV–effect of residual variable the explanatory variables.

**Fig 3:**
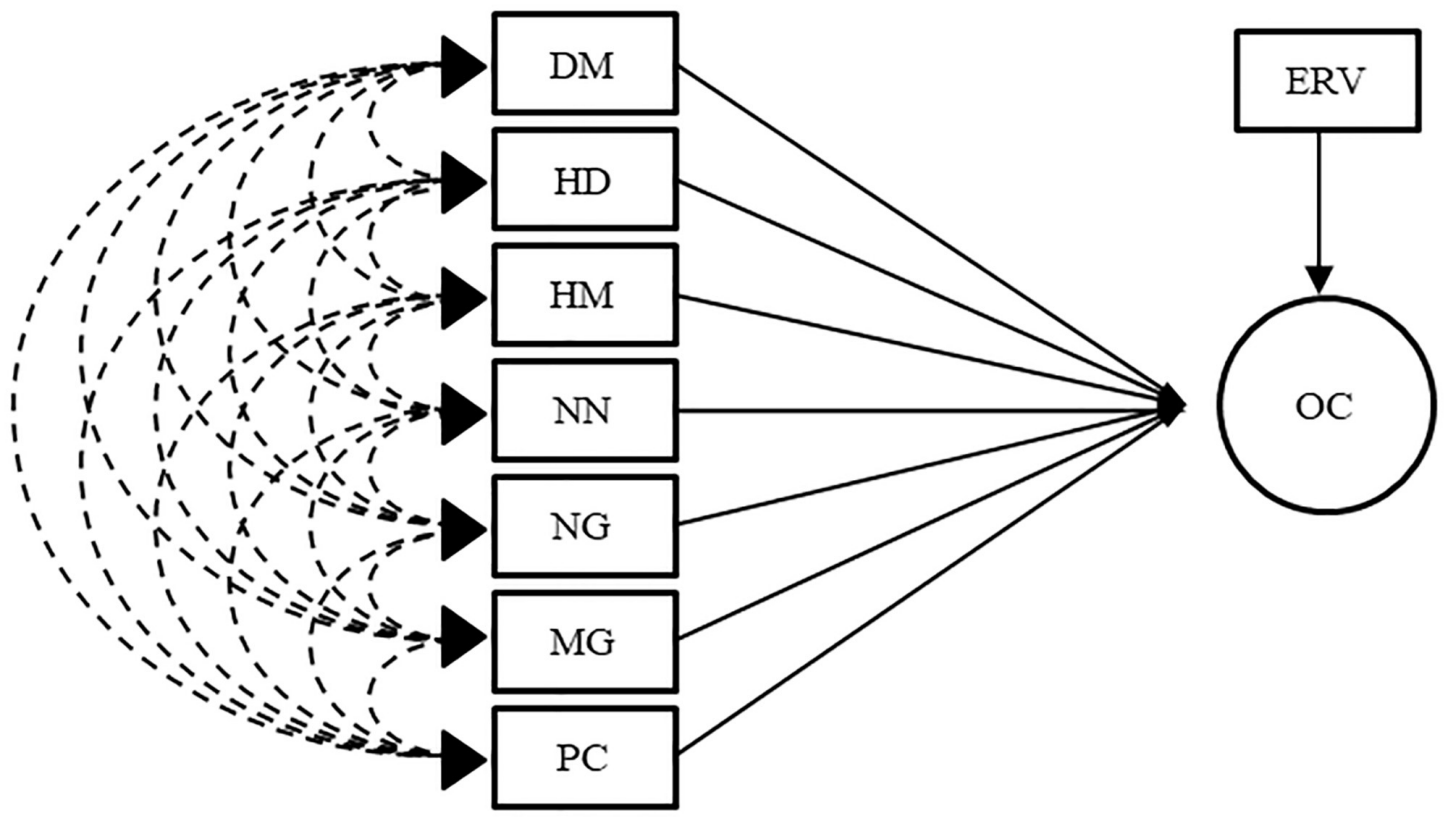
Path analysis diagram for matrices R_2_ and R_2_s’s. Oil content was considered the main trait DM—days to maturity; HD—hypocotyl diameter; HM—height of plants on maturity; NN—number of nodes in the main stem per plant; NG—number of grains per plant; MG—mass of grains per plant; PC—protein content in grains; OC—oil content in grains and ERV–effect of residual variable the explanatory variables.

Two procedures were used to address the problems caused by collinearity. Firstly, the path analysis through the ridge regression onto matrix R (Fig 1) as proposed by [16, 17] and secondly, traditional path analysis after eliminating traits which showed high correlation with another (trait culling). For the path analysis with trait culling, two new correlation matrices were obtained: R_1_ and R_2_ by the elimination of different traits in each matrix, and the traditional path analysis were run as shown in the diagrams (Figs 2 and 3, respectively). The traits eliminated were chosen considering its correlation with other traits kept in the matrix and how often they are evaluated in soybean breeding programs, for example: HF and HM are highly correlated (0.92) and HF is less frequently measured in soybean breeding. Herewith, the reduction of collinearity was expected.

To establish the constant value K, the direct effects were plotted in a graph as a function of the values of K. The minimum value of K that provided stable estimates was chosen to run the analysis, as recommended by [12].

### Simulation of a new dataset and validation of the path analysis procedures

To validate the results obtained with the experimental data, one hundred of new populations composed of 100 individuals were simulated using the means and covariance matrix of the original dataset in order to keep biological sense in the variables. The large number of simulated populations of few individuals avoids the reproduction of results obtained with original dataset.

Then, one correlation matrix was estimated for each population. After obtained the correlation matrices for each population the same procedures of path analysis were adopted to the simulated dataset. The same set of variables and procedures performed for R, R_1_ and R_2_ were also performed respectively for simulated matrices Rs’s, R_1_s’s and R_2_s’s.

The best linear unbiased predictions (BLUPs) of the genotypes and variance components were estimated using the software SELEGEN-REML/BLUP [31]; and the correlation coefficients, path analysis, and multicollinearity diagnosis were performed for real data using the software GENES [33] and for simulated data using R software 3.5.3 [34].

## Results

Significant genetic variability was found for all vegetative, reproductive and grain quality traits. The lowest and the greater heritability (h²) estimate were found for OC (0.368) and DF (0.992) respectively (Table 2). Grain quality traits (OC and PC) and MG showed lower heritability estimated than most of traits. Accuracy (r_âa_) values varied from 0.854 for OC to 0.992 for DF. The experimental coefficients of variation (CVe%) varied from 2.81 for PC to 20.3 for NS. Genotypic coefficient of variation (CVg%) ranged from 32.1 for HF to 3.00 for PC and CVg% values were greater than CVe% for all traits. The relative coefficient of variation, which is the ratio between the CVg% and CVe% ranged from 20.3 for HF and 2.81 for NP.

**Table 2:** Genetic and environmental parameters estimated for days to flowering (DF), height of plants on flowering (HF), days to maturity (DM), hypocotyl diameter (HD), height of plants on maturity (HM), number of pods per plant (NP), number of nodes in the main stem per plant (NN), number of branches per plant (NB), number of grains per plant (NG), mass of grains per plant (MG), oil content in grains (OC), and protein content in grains (PC) evaluated in 15 soybean genotypes.

Parameters	DF	HF	DM	HD	HM	NP	NN	NB	NG	MG	OC	PC
σ²_g_^†^	34.3 *	201 *	90.2 *	0.333 *	174 *	52.5 *	1.84 *	0.486 *	151 *	2.85 *	0.667 *	1.38 *
σ²_e_	2.81	24.4	28.5	0.228	71.9	10.6	0.544	0.832	65.4	1.79	1.24	1.21
σ²_f_	37.1	224	119	0.561	246	63.2	2.39	1.32	217	4.64	1.91	2.58
h^2^_g_	0.924	0.891	0.76	0.593	0.708	0.832	0.772	0.368	0.698	0.613	0.35	0.532
r_âa_	0.992	0.988	0.97	0.938	0.961	0.98	0.972	0.863	0.959	0.942	0.854	0.922
CVg%	14.09	32.1	8.07	11.5	19.5	23.9	11.206	15.5	18.4	14.8	4.28	3
CVe%	4.03	11.2	4.53	9.54	12.5	10.8	6.091	20.3	12.1	11.7	5.84	2.81
CVr	3.49	2.86	1.78	1.21	1.56	2.22	1.84	0.764	1.52	1.26	0.733	1.07
PEV	0.552	4.76	5.36	0.04	13.3	2.04	0.103	0.124	12	0.319	0.181	0.206
SEP	0.743	2.18	2.31	0.2	3.64	1.43	0.321	0.352	3.47	0.565	0.425	0.453
Mean	41.5	44.1	118	5.01	67.8	30.3	12.1	4.49	66.8	11.4	19.1	39.1

* Significant at the 0.01 probability level by the LRT test (Likelihood Ratio Test) using chi-squared test with one degree of freedom;

^**†**^σ²_g_ = genotypic variance; σ²_e_ = residual variance; σ²_f_ = individual phenotypic variance; h^2^_g_ = wide hereditability of individual plots; r_âa_ = selection accuracy of genotypes, assuming no plots lost; CVg% = genotypic coefficient of variation; CVe% = residual coefficient of variation; CVr = CVg/CVe = relative coefficient of variation; PEV = prediction error variance, assuming no plots lost; SEP = standard deviation of predicted genetic values, assuming no plots lost and Mean = general mean of three experiment.

Genotypic values prediction for each genotype is presented in Table 3 and correlation coefficients estimated between genetic values are presented in Table 4. The correlation coefficients indicates that MG is high influenced by DF, DM, NN, and NP. Besides this, MG was more correlated to NP (0,79). With the respect to the oil content, NS showed the greater correlation with OC (0.75). The oil content was also strongly correlated with the yield components NN (0.64), NP (0.65), NG (0.67) and also with MG (0.67). High and significant correlations were found for number of pods (NP) and NN (0.88), and for NN and DM (0.86). Regarding the traits of grain quality, a high and negative correlation were found for PC × NS (-0.56), PC × NG (-0.53), and PC × OC (-0.60). The means of the correlation coefficients from the 100 simulated populations showed to be very similar to those estimated from the original dataset as also shown in Table 4.

**Table 3:** Genotypic values (u + g) of the genotypes evaluated for days to flowering (DF), height of plants on flowering (HF), days to maturity (DM), hypocotyl diameter (HD), height of plants on maturity (HM), number of nodes in the main stem per plant (NN), number of pods per plant (NP), number of branches per plant (NB), number of grains per plant (NG), mass of grains per plant (MG), oil content in grains (OC), and protein content in grains (PC) evaluated in 15 soybean genotypes.

Variety	Traits											
	DF	HF	DM	HD	HM	NN	NP	NB	NG	MG	OC	PC
	u + g											
BMX Apolo RR	36.0	21.4	106.0	4.5	45.4	20.9	10.1	3.5	54.2	9.0	17.9	41.2
DM 5958 IPRO	34.4	28.2	106.2	4.0	51.2	23.4	10.9	4.4	54.1	9.1	19.4	38.4
NA 5909 RR	35.2	42.7	107.4	4.6	72.4	26.1	10.5	4.9	60.0	10.7	18.8	38.6
TMG 7062 IPRO	36.7	35.6	103.5	4.7	59.5	22.1	10.9	4.1	53.0	10.9	18.9	38.8
DM 6563 IPRO	36.7	30.6	114.1	4.8	61.2	26.6	11.9	3.7	64.5	10.9	19.0	38.0
BMX Potência RR	37.1	29.7	111.3	4.7	59.9	24.0	11.3	4.2	58.6	9.7	18.3	39.6
BMX Desafio RR	34.3	30.8	117.3	5.6	62.7	23.8	11.7	3.9	58.2	10.8	18.1	40.2
M 7739 IPRO	45.4	50.5	119.4	5.5	66.5	33.1	12.3	5.0	76.4	12.5	19.5	37.6
P98Y30 RR	44.8	56.7	123.3	5.0	79.9	37.1	13.6	5.5	80.1	12.9	20.6	38.8
M8349 IPRO	45.4	46.0	124.3	5.1	63.7	35.5	12.6	5.0	82.6	13.9	19.8	37.8
TMG 132 RR	47.4	52.0	128.8	4.8	70.4	36.5	13.3	5.1	80.3	11.2	19.1	38.8
M 9056 RR	51.2	65.1	127.3	5.8	94.1	42.6	15.0	5.0	88.4	13.4	19.5	38.2
M 8221 RR	46.2	50.7	119.7	5.8	69.2	40.8	12.5	4.5	66.8	12.3	19.0	40.3
P98N71	45.8	57.1	131.2	4.9	76.3	31.1	13.1	4.5	61.0	10.9	19.3	39.8
Conquista	46.3	64.0	125.7	5.5	84.6	31.0	12.1	4.1	63.6	13.4	19.3	40.1

u + g = general mean summed to genotypic effects for each genotype.

**Table 4:** Estimates of genotypic correlation between the traits DF, HF, DM, HD, HM, NP, NN, NB, NG, MG, OC and PC for fifteen soybean genotypes from the original data set below the diagonal and for the simulated populations above the diagonal, including the standard deviation bellow.

Traits	DF	HF	DM	HD	HM	NP	NN	NB	NG	MG	OC	PC
DF	1	0.9	0.86	0.61	0.75	0.91	0.86	0.61	0.81	0.78	0.59	-0.19
		0.02	0.03	0.06	0.04	0.02	0.03	0.06	0.04	0.04	0.07	0.1
HF	0.9**	1	0.82	0.6	0.92	0.83	0.8	0.65	0.68	0.8	0.65	-0.2
			0.03	0.06	0.01	0.03	0.03	0.06	0.05	0.04	0.07	0.1
DM	0.86**	0.82**	1	0.59	0.72	0.79	0.86	0.51	0.71	0.69	0.51	-0.1
				0.07	0.05	0.05	0.03	0.08	0.05	0.06	0.09	0.11
HD	0.62*	0.61*	0.59*	1	0.62	0.63	0.58	0.22	0.5	0.73	0.14	0.06
					0.06	0.06	0.08	0.1	0.07	0.05	0.11	0.1
HM	0.75**	0.92**	0.73**	0.63*	1	0.73	0.79	0.56	0.62	0.73	0.53	-0.19
						0.04	0.03	0.07	0.05	0.04	0.08	0.1
NP	0.91**	0.83**	0.78**	0.65**	0.73**	1	0.88	0.72	0.87	0.78	0.65	-0.29
							0.03	0.05	0.02	0.04	0.06	0.09
NN	0.86**	0.80**	0.86**	0.59*	0.79**	0.88**	1	0.64	0.85	0.7	0.64	-0.33
								0.06	0.03	0.06	0.07	0.09
NB	0.62*	0.66*	0.51*	0.24	0.58*	0.72**	0.65**	1	0.77	0.56	0.76	-0.57
									0.04	0.06	0.04	0.07
NG	0.81**	0.68*	0.72**	0.52*	0.62*	0.87**	0.85**	0.77**	1	0.77	0.66	-0.54
										0.04	0.06	0.07
MG	0.78**	0.81**	0.69**	0.73**	0.73**	0.79**	0.70**	0.57**	0.77**	1	0.67	-0.36
											0.06	0.09
OC	0.60*	0.66*	0.51	0.16	0.54*	0.65**	0.64**	0.75**	0.67**	0.67**	1	-0.6
												0.06
PC	-0.19	-0.2	-0.09	0.05	-0.19	-0.28	-0.33	-0.56*	-0.53*	-0.36	-0.60*	1

* Significant at the 0.05 probability level;

** Significant at the 0.01 probability level according to Mantel’s test.

Multicollinearity diagnosis analysis showed severe multicollinearity for matrix R; moderate, but very close to the limit to be weak for matrix R_1_ (not contemplating the traits DF, HF and NB); and weak for matrix R_2_ (not contemplating the traits DF, HF, NN and NB) (Table 5).

**Table 5:** Eigenvalues, condition number (CN) and its standard deviations between parenthesis of the correlation matrices R, R_1_, R_2_, Rs, R_1_s and R_2_s between explanatory variables.

Rank	Eigenvalues					
	R^†^	R_1_	R_2_	Rs	R_1_s	R_2_s
1	7.67	5.13	4.72	7.65(0.28)	5.46(0.21)	4.61(0.20)
2	1.40	1.36	1.11	1.44(0.16)	1.19(0.11)	1.19(0.11)
3	0.590	0.550	0.388	0.618(0.09)	0.534(0.09)	0.523(0.09)
4	0.454	0.338	0.336	0.444(0.06)	0.336(0.05)	0.317(0.05)
5	0.314	0.282	0.240	0.302(0.05)	0.203(0.03)	0.185(0.03)
6	0.226	0.183	0.128	0.215(0.03)	0.150(0.03)	0.121(0.02)
7	0.147	0.111	0.077	0.139(0.02)	0.075(0.01)	0.050(0.01)
8	0.104	0.048		0.096(0.01)	0.047(0.01)	
9	0.058			0.054(0.01)		
10	0.037			0.033(0.01)		
11	0.004			0.003(0.00)		
CN	2081	105	86.5	2337(559.91)	119(22.53)	95.3(18.22)
Multicollinearity level	Severe	Moderate	Weak	Severe	Moderate	Weak

^**†**^R and Rs = original set of the correlation matrix between explanatory variables from original and simulated dataset, respectively; R_1_ and R_1_s = correlation matrix with trait culling to three explanatory variables from original and simulated dataset, respectively; R_2_ and R_2_s = correlation matrix with trait culling to four explanatory variables from original and simulated dataset, respectively.

There was no direct or indirect effect higher than the residual effect (0.585) and the determination coefficient was not satisfactory (0.657) for matrix R (Table 6). For the R_1_ matrix, path analysis showed high determination coefficient (0.856) and the effect of the residual variable was outdone by direct effects of traits on OC (Table 7). The traits DM, HD, HM, and NG presented direct effects with sign opposite to the correlation coefficient in analysis of the matrix R_1_. A weak association of DM and HM with OC was observed. On the other hand, NP, NN, and MG were noticed to be highly associated with OC by the direct effects 0.427, 0.632 and 0.967.

**Table 6:** Estimates of direct and indirect effects for: DF, HF, DM, HD, HM, NP, NN, NB, NG, MG and PC on OC evaluated in 15 soybean genotypes onto the matrix R.

	Explanatory variables										
Effect	DF	HF	DM	HD	HM	NP	NN	NB	NG	MG	PC
Direct on OC	0.035	0.141	0.019	-0.207	0.020	0.086	0.105	0.196	0.022	0.216	-0.205
Indirect via DF	-	0.032	0.031	0.022	0.027	0.032	0.031	0.022	0.029	0.028	-0.007
Indirect via HF	0.127	-	0.115	0.086	0.131	0.117	0.113	0.094	0.096	0.114	-0.029
Indirect via DM	0.017	0.016	-	0.011	0.014	0.015	0.016	0.010	0.014	0.013	-0.002
Indirect via HD	-0.128	-0.126	-0.122	-	-0.129	-0.134	-0.121	-0.049	-0.106	-0.151	-0.010
Indirect via HM	0.015	0.018	0.014	0.012	-	0.014	0.016	0.011	0.012	0.014	-0.004
Indirect via NP	0.079	0.071	0.068	0.056	0.063	-	0.076	0.063	0.075	0.068	-0.024
Indirect via NN	0.090	0.084	0.090	0.061	0.083	0.092	-	0.068	0.089	0.073	-0.034
Indirect via NB	0.121	0.130	0.101	0.046	0.113	0.142	0.126	-	0.151	0.111	-0.109
Indirect via NG	0.018	0.015	0.016	0.012	0.014	0.019	0.019	0.017	-	0.017	-0.012
Indirect via MG	0.169	0.174	0.148	0.158	0.158	0.170	0.152	0.122	0.167	-	-0.077
Indirect via PC	0.039	0.041	0.018	-0.010	0.040	0.057	0.067	0.114	0.108	0.073	-
Total^†^	0.60	0.66	0.51	0.16	0.54	0.65	0.64	0.76	0.67	0.67	-0.60
R²	0.657										
K	0.445										
ERV	0.585										

^**†**^Total = correlation coefficient between the trait and OC; R² = Determination coefficient; K = constant used in the analysis and; ERV = effect of the residual variable.

**Table 7:** Estimates of direct and indirect effects for: DM, HD, HM, NP, NN, NG, MG and PC on OC evaluated in 15 soybean genotypes onto the matrix R_1_.

	Explanatory variables^††^							
Effect	DM	HD	HM	NP	NN	NG	MG	PC
Direct on OC	-0.126	-0.758	-0.110	0.427	0.632	-0.556	0.967	-0.223
Indirect via DM	-	-0.075	-0.092	-0.099	-0.109	-0.091	-0.087	0.011
Indirect via HD	-0.449	-	-0.474	-0.491	-0.445	-0.391	-0.556	-0.037
Indirect via HM	-0.080	-0.069	-	-0.081	-0.087	-0.069	-0.081	0.021
Indirect via NP	0.333	0.276	0.312	-	0.374	0.371	0.335	-0.119
Indirect via NN	0.543	0.371	0.500	0.554	-	0.536	0.443	-0.207
Indirect via NG	-0.398	-0.286	-0.347	-0.483	-0.472	-	-0.429	0.294
Indirect via MG	0.663	0.709	0.709	0.760	0.679	0.747	-	-0.344
Indirect via PC	0.020	-0.011	0.043	0.062	0.073	0.118	0.079	-
Total^†^	0.51	0.16	0.54	0.65	0.64	0.67	0.67	-0.60
R²	0.856							
ERV	0.379							

^**†**^Total = correlation coefficient between the trait and OC; R² = determination coefficient; and ERV = effect of the residual variable.

^**††**^ The traits DF, HF and NB were eliminated from the matrix R in originating the matrix R_1_.

Strong direct effects of NN (0.804) and MG (1.05) on OC were found (Table 8). The ERV (0.410) and R² (0.832) found for path analysis on matrix R_2_ fitted better than the analysis of the matrix R, but not as well as the analysis of the matrix R_1_. Analysis of the matrix R_2_ revealed results very similar to those of matrix R_1_.

**Table 8:** Estimates of direct and indirect effects for: DM, HD, HM, NN, NG, MG, PC on OC evaluated in 15 soybean genotypes onto the matrix R_2_.

	Explanatory variables^††^						
Effect	DM	HD	HM	NN	NG	MG	PC
Direct on OC	-0.158	-0.744	-0.095	0.804	-0.347	1.05	-0.148
Indirect via DM	-	-0.094	-0.115	-0.136	-0.113	-0.018	0.014
Indirect via HD	-0.440	-	-0.466	-0.437	-0.384	-0.546	-0.036
Indirect via HM	-0.069	-0.059	-	-0.075	-0.059	-0.070	0.018
Indirect via NN	0.690	0.472	0.636	-	0.682	0.564	-0.260
Indirect via NG	-0.249	-0.179	-0.217	-0.295	-	-0.268	0.184
Indirect via MG	0.718	0.768	0.768	0.735	0.809	-	-0.372
Indirect via PC	0.013	-0.007	0.029	0.048	0.079	0.054	-
Total^†^	0.51	0.16	0.54	0.64	0.67	0.67	-0.60
R²	0.832						
ERV	0.410						

^**†**^Total = correlation coefficient between the trait and OC; R² = determination coefficient; and ERV = effect of the residual variable.

^**††**^ The traits DF, HF, NN and NB were eliminated from the matrix R in originating the matrix R_2_.

The path analyses on Rs’s matrices revealed a lower average k-value (0.334) and a higher determination coefficient (0.701) as presented in Table 9. The same promising traits (NP, NN and MG) for indirect selection in matrices R_1_ were also appointed by path analysis on R_1_s’s matrices whose average estimates are shown in Table 9. The average condition number estimated for simulated populations were slightly greater than those estimated for the original dataset.

**Table 9:** Mean direct effects and standard deviation between parentheses for explanatory traits, k-value (K), effect of residual variable (ERV) and R-squared (R²) of the path analysis for the simulated populations.

	DF	HF	DM	HD	HM	NP	NN	NB	NG	MG	PC	K	ERV	R²
Rs’s^†^	0.011	0.169	0.016	-0.265	-0.013	0.099	0.140	0.211	-0.024	0.283	-0.203	0.334	0.544	0.701
	(0.04)	(0.05)	(0.04)	(0.09)	(0.07)	(0.04)	(0.07)	(0.04)	(0.08)	(0.1)	(0.04)	(0.14)	(0.05)	(0.04)
R_1_s’s	-	-	-0.124	-0.772	-0.106	0.416	0.631	-	-0.552	0.969	-0.214	-	0.363	0.866
	-	-	(0.10)	(0.10)	(0.07)	(0.11)	(0.16)	-	(0.12)	(0.11)	(0.07)	-	(0.04)	(0.03)
R_2_s’s	-	-	-0.146	-0.762	-0.091	-	0.803	-	-0.357	1.048	-0.143	-	0.394	0.844
	-	-	(0.10)	(0.10)	(0.08)	-	(0.16)	-	(0.12)	(0.11)	(0.07)	-	(0.04)	(0.03)

^**†**^Rs’s = matrices from simulated dataset composed of DF, HF, DM, HD, HM, NP, NN, NB, NG, MG and PC; R_1_s’s = matrices from simulated dataset composed of DM, HD, HM, NP, NN, NG, MG and PC; R_2_s’s = matrices from simulated dataset composed of DM, HD, HM, NN, NG, MG and PC.

## Discussion

### Genetic control and variance components

The genetic variability noticed between soybean cultivars shows that they can be used as genitor in breeding programs. The variability between genitors is fundamental to generate segregating populations that provide genetic gains [7].

Low heritability and accuracy were expected for mass of grains and quality traits, since they are controlled by a larger number of genes and are more affected by environmental factors [5, 6]. The values of accuracy found in this research are considered high according to [35]. These results indicate good experimental quality; thus, the data set and data analysis can be used in trustworthy inferences by researchers.

According a classification proposed by [36] the residual coefficients of variation (CVe%) were classified as low which indicates good experimental quality. The greatest value of CVe% was found for number of branches. According to [37] soybean plants have the ability to adjust the number of branches to compensate for the distance from other plants and intercept the maximum amount of light. The authors named this phenotypic plasticity. Despite equal spacing between pots, randomizing the treatments inside the blocks induced different levels of competition to the same genotype in different blocks. This effect was accounted for the environmental effects, therefore, decreases the coefficient of variance.

According to [38] an statistic usually employed on genetic evaluations is the ratio between the genetic coefficient of variation and the experimental coefficient of variation which is called relative coefficient of variation (CVr) and is related to experimental accuracy. Estimates of relative CVr higher than 1 indicate more accurate and precise inferences about the rank of the genotypes, which was found for 10 traits with the exception of NB and OC.

### Genetic correlation among traits and multicollinearity diagnosis

The traits days to flowering, days to maturing, number of nodes and number of pods were found strongly correlated to mass of grains. These results corroborate with results for genetic correlation found by [39].

The strong correlation between oil content and grain yield found is reinforced by [40]. The high correlation of number of nodes and number of pods with grains yield are reported in the literature as well as found in this research [24, 39]. This is due to the grains yield comes from pods which are developed in axillary buds located on plant stems or branches originated from the nodes. Besides this, the high correlation between number of nodes and days to maturing shows that late maturing genotypes tend to present higher grain yield per plant. These results agree with those of [15].

A strong and negative correlation between oil and protein content were also found by [3, 41, 42] in molecular markers studies. [43] suggested that both traits are controlled by the same gene or group of genes, which were later reported as a major QTL in the crhomossome 20 by [44].

The elimination of traits that are strongly correlated and have biological interpretations such as DF and DM or HF and HM was shown to be effective to overcome multicollinearity. Thus, matrix R_1_ or R_2_ each contains just one of these traits from these pairs. This discussion is supported by the results of simulated data presented in Table 4 that showed very similar results for 100 populations. However the matrices comprised of the same set of variables presented the same level of collinearity problems.

### Path analysis results and comparison among procedures

The poor adjustment of the ridge path analysis onto R and Rs’s matrices and the greater effects of the residual variable in comparison with the direct effects, leads us to affirm that the estimates are not reliable (Tables 6 and 8). [15] obtained direct effect estimates higher than 1 for two traits in a traditional path analysis on a correlation matrix with strong collinearity problems for soybean. The authors also ran a ridge analysis, which gave estimates between 0 and 1, and these two traits did not show relevant direct effects. The K value used by the authors was of 0.05, whereas the K value needed to overcome collinearity in our original and simulated dataset were much greater. [12] affirm that high K values lead to higher bias in the regression and may provide results without biological mean.

High values for effects of residual variable (ERV) may be found when the number of explanatory variables is not enough to explain variation of the main trait. However, the estimates for matrix R_1_ showed that even after reducing the number of explanatory variables, it was possible to explain a large part of the total variance. This result was also observed by [14] when they found greater regression adjustment after trait culling of the explanatory variables. [10] affirm that the exclusion of variables allows estimating path coefficients with biological meaning, what is an advantage of trait culling.

A weak association of HM with OC was observed by the low direct and indirect effects, although HM showed significant correlation with OC. Likewise, a high correlation between PC and OC and weak direct effect was noticed. These results proves that the correlation coefficient does not necessarily give the best idea of association between variables and researches need to be careful at choose traits for indirect selection based just on correlation coefficient. On the other hand, NP, NN and MG are recommended to be used in indirect selection for OC, since the magnitude of direct effects were close to the correlation, which is the sum of direct and indirect effects involving each trait. In addition, these traits are easier, cheaper, and quicker to measure than laboratory procedures to measure OC. Besides this, NP and NN may be evaluated before complete maturation, which allows earlier selection, and MG is routinely evaluated in soybean experiments. In order to achieve genetic gains on OC we also recommend the use of a selection index including the traits that showed high direct effects on OC.

The direct effect of MG on OC from path analysis on R_2_ matrix was 1.05, although estimates greater than 1 were not expected, once the matrix R_2_ showed weak collinearity problems. The path analysis on R_2_s’s matrices also presented means direct effects of MG on OC higher than 1. The strong and positive direct effects of NN and MG on OC showed in R_1_ analysis were confirmed by path analysis on R_2_ and R_2_s’s matrices and encourage researches to use them for indirect selection. The same discussion mentioned above regarding PC in matrix R_1_ can be applied to matrix R_2_.

Although the matrix R_1_ showed moderate to strong problems with collinearity according to [45], the path analysis onto this set of variables was the most suitable for this study, since it provided the greatest determination coefficient of the regression, the lowest effect of the residual variable, and all effects were estimated inside the parameter space, which ranges from -1 to 1 for direct, indirect and residual variable effects. These results corroborate with those found by [14] when the authors tested three methods of path analysis (traditional, ridge path analysis and trait culling) and concluded that the trait culling provided a fit statistics more accurate. The discussions about the quality of the path analysis onto R_1_ matrix were validated by path analysis onto R_1_s’s matrices according to the great regression adjustment (0.844) and the high effect of the residual variable. Furthermore, the choice of the R_1_ matrix allows considering one more variable in the study (NP).

The ridge path analysis was not an appropriated approach for overcome the collinearity problem, since a low R² (0.657) and high ERV (0.585) were found. Besides of this, no direct effects greater than ERV were found for analysis onto matrices R and R_s’s_ (Tables 6 and 8). Path analysis after trait culling for matrices R_1_ and R_2_ showed to be the best strategy for overcome collinearity problem since it presented a well-fit of the regression denoted by the values of R² (0.856 and, 0.832 respectively) and direct effects greater than the ERV (0.379 and 0.410, respectively). The results found onto simulated matrices supported these findings (Table 9). In addition, trait culling is a simple technique and allows to the researcher exclude the variable that contributes more to the collinearity considering its biological relationship with the main trait.

## Conclusions

There is a strong relation of causation of mass of grains per plant, number of nodes, and number of pods with the oil content in grains and they could be used for indirect selection. Trait culling was more suitable and simpler strategy than ridge path analysis to overcome collinearity problems.

Eliminate one between two similar traits biologically was efficient to reduce condition number and, consequently, overcome multicollinearity.

## Supporting information

S1 Appendix Maintainer company, cultivar name and to National Cultivar Registration (RNC) register number of 15 commercial soybean cultivars used for the research. (DOCX)
