# Supplementary material for: Overcoming collinearity in path analysis of soybean [Glycine max (L.) Merr.] grain oil content
Source: PLoS One. 2020 May 22;15(5):e0233290. doi: 10.1371/journal.pone.0233290 (PMC7244132; doi:10.1371/journal.pone.0233290)
Supplement: S1 Appendix — (DOCX) [file pone.0233290.s001.docx]

S1 Appendix – Maintainer Company, Cultivar name and to National Cultivar Registration (RNC) register number of 15 commercial soybean cultivars used for the research.

| Maintainer Company | Cultivar | RNC register number |
| --- | --- | --- |
| Dupont do Brasil S/A - Divisão Pioneer Sementes | P98N71 | 12507 |
|  | P98Y30 RR | 26144 |
| Empresa Brasileira de Pesquisa Agropecuária - EMBRAPA/ Fundação de Apoio à Pesquisa Agropecuária de Mato Grosso - Fundação MT | MG/BR 46 - Conquista | 1209 |
| GDM Genética do Brasil S.A. | BMX Apolo RR (Dmario 58i) | 21599 |
|  | BMX Desafio RR (8473 RSF) | 28779 |
|  | BMX Potência RR | 22323 |
|  | DM 5958 IPRO | 29553 |
|  | DM 6563 IPRO | 29560 |
| Monsoy LTDA | M 8221 RR | 22583 |
|  | M 9056 RR | 19394 |
|  | M7739 IPRO | 29692 |
|  | M8349 IPRO | 29779 |
| NIDERA Seeds Brasil LTDA | NA 5909 RR | 24590 |
| TMG Tropical Melhoramento e Genética LTDA | TMG 132 RR | 22710 |
|  | TMG 7062 IPRO | 31288 |
